# Expression and Prognostic Value of PIK3CA, VEGF, IL-8, IL-10, and RIP2 in Diffuse Large B-Cell Lymphoma

**DOI:** 10.1155/2022/2637581

**Published:** 2022-12-07

**Authors:** Na Shen, Yanfang Yu, Ruiying Zhang, Ying Guo, Mei Liu, Mengtian Tan, Jianxia Liu, Jing Bai, Li Li, Keyu Liu, Ran Wang, Jinglan He

**Affiliations:** ^1^Department of Hematology, Affiliated Hospital of Hebei Engineering University, Handan, Hebei, China; ^2^Department of Science and Education, Affiliated Hospital of Hebei Engineering University, Handan, Hebei, China; ^3^Clinical School of Medicine, Hebei University of Engineering, Handan, Hebei, China; ^4^Department of Quality Control, Affiliated Hospital of Hebei Engineering University, Handan, Hebei, China; ^5^Department of Geriatrics, Affiliated Hospital of Hebei Engineering University, Handan, Hebei, China; ^6^Bone Marrow Laboratory, Affiliated Hospital of Hebei Engineering University, Handan, Hebei, China; ^7^Department of Emergency, Affiliated Hospital of Hebei Engineering University, Handan, Hebei, China; ^8^Department of Orthopedics, Affiliated Hospital of Hebei Engineering University, Handan, Hebei, China

## Abstract

**Background:**

To explore clinical features and prognostic value of vascular endothelial growth factor (VEGF), interleukin (IL) 8, IL-10, phosphatidylinositol-4,5-bisphosphate 3-kinase catalytic subunit alpha (PIK3CA), and receptor-interacting protein-2 (RIP2) in diffuse large B-cell lymphoma (DLBCL).

**Methods:**

A total of 68 DLBCL patients admitted to the Affiliated Hospital of Hebei Engineering University from January 2017 to June 2021 were included in this retrospective analysis. Serum VEGF was detected by enzyme-linked immunosorbent assay, serum IL-8 and IL-10 were detected by chemiluminescent enzyme immunoassay, and expression of PIK3CA and RIP2 in tumors was detected by immunohistochemistry. The correlation between clinical features of DLBCL and tumor-related index were analyzed. Cox regression was conducted to explore risk factors and hazard ratio.

**Results:**

The serum level or expressions of VEGF, IL-8, IL-10, and RIP2 were significantly elevated with the increase of Ann Arbor Stage, International Prognostic Index (IPI) scores, Eastern Cooperative Oncology Group (ECOG) scores, serum lactate dehydrogenase (LDH) level, and the number of extranodal sites (all *P* < 0.05). Beside, these serum indexes were significantly higher in patients with the presence of extranodal involvement and germinal center B-cell (GCB), but significantly lower in patients with the presence of bone marrow involvement (all *P* < 0.05). Cox regression analysis for overall survival revealed that high expression of VEGF, high level of serum IL-8, serum IL-10, and RIP2, Ann Arbor Stage (III-IV), number of extranodal sites (>1), serum LDH level (≥245 U/L), IPI scores (3–5), ECOG scores (≥2), and bone marrow involvement were independent risk factors for the prognosis of DLBCL patients (all *P* < 0.05).

**Conclusion:**

The serum levels of VEGF, IL-8, and IL-10, as well as the expression of RIP2 and PIK3CA in tumor tissues, were highly correlated to clinical features of DLBCL, and high expression level of these indexes may have adverse effects for the prognosis of DLBCL patients.

## 1. Introduction

Non-Hodgkin lymphoma is one of the most highly heterogeneous malignant neoplasms in the lymphohematopoietic system, with a 12th place of incidence and a 10th place of mortality among malignant neoplasms [1, 2]. Diffuse large B-cell lymphoma (DLBCL), the most common type of non-Hodgkin lymphoma, is extremely aggressive and highly heterogeneous in terms of clinical manifestations, epidemiology, and prognosis, and its incidence accounts for 31–34% of non-Hodgkin lymphomas and generally >40% in Asian countries [3]. International prognostic index (IPI) scores are commonly used to evaluate the prognosis of DLBCL patients, but they are still inaccurately assessed for some patients [4]. Therefore, an indicator with specificity and sensitivity is important for disease monitoring and prognosis evaluation in non-Hodgkin lymphoma.

Phosphatidylinositol-4,5-bisphosphate 3-kinase catalytic subunit alpha (PIK3CA) is an oncogene that positively regulates the PI3K, Akt/mTOR signaling pathway, and the activation of this signaling pathway is involved in the progression of many malignancies, such as colon, breast, lung, and lymphoma. However, it has received little attention in DLBCL [5]. Vascular endothelial growth factor (VEGF) is a cytokine that stimulates mitogenesis of vascular endothelial cells and binds to VEGF receptors to promote tumor vascularization and plays a vital role in tumor proliferation, infiltration, metastasis, and prognosis [6]. Interleukin (IL) 8 and IL-10 play a crucial role in the growth, differentiation, and maturation of B lymphocytes and are involved in immune regulation, tumorigenesis, and growth [7, 8]. Receptor-interacting protein-2 (RIP2) is an important regulator for maintaining protein stability and initiates intrinsic immune responses through Nod1 and Nod2 ligands. Moreover, RIP2 is involved in the induction of apoptosis and activation of the NF-*κ*B pathway, which plays an important role in the pathogenesis of DLBCL [9]. However, there are few reports that explored these indexes on the prognosis of DLBCL.

Thus, this study was designed to investigate the expression and prognostic value of PIK3CA, VEGF, IL-8, IL-10, and RIP2 in DLBCL.

## 2. Materials and Methods

The study protocol complied with the relevant requirements of the World Medical Association Declaration of Helsinki and was approved by the ethics committee of Affiliated Hospital of Hebei Engineering University (No. 2018[K]061). Informed consent was obtained from all the study subjects before enrollment.

### 2.1. Patients

This retrospective study enrolled 68 patients with DLBCL admitted to our hospital from January 2017 to June 2021. Patients meeting the following criteria were included: (I) diagnosed according to the Chinese Guidelines for the Diagnosis and Treatment of DLBCL (2013 version) [10] and (II) initially diagnosed. Patients meeting the following criteria were excluded: (I) with previous alcoholism or drug addiction; (II) with complications of the heart, liver, spleen, lung, kidney, and other organs; (III) with family genetic diseases; (IV) a second malignancy; and (V) incomplete clinical and pathological data.

The following characteristics of patients were included: age of DLBCL onset, gender, Ann Arbor Stage, B symptoms, the number of extranodal sites, serum level of lactate dehydrogenase (LDH), IPI scores, Eastern Cooperative Oncology Group (ECOG) scores, bone marrow involvement, extranodal involvement, and pathological types (Hans).

### 2.2. Detection of Serum VEGF, IL-8, and IL-10 Levels

Totally 5 mL of fasting venous blood of DLBCL patients was used for testing before treatment. VEGF was determined by the enzyme-linked immunosorbent assay (Ruihua Medical Technology Development Co., Ltd., Fuzhou, China), and IL-8 and IL-10 were determined by chemiluminescent enzyme immunoassay (Ruihua Medical Technology Development Co., Ltd., Fuzhou, China).

### 2.3. Immunohistochemistry (IHC)

Tissue specimen sections were placed in a 65°C oven for 1 h, dewaxed in xylene, dehydrated in gradient alcohol, and rinsed 3 times. According to the instruction of the immunohistochemistry kit (Jiehui BOGAO Biotechnology Co., Ltd., Beijing, China), sections were incubated in 3% hydrogen peroxide solution for 10 min under dark conditions and washed by phosphate buffer saline (PBS), and antigen was repaired and incubated with a PIK3CA primary antibody or RIP2 (Abcam Co., Ltd, England; number: ab155529) primary antibody with a resolution of 1 : 200, thereafter incubated with a secondary antibody. Then, the sections were washed by PBS again, color developed using diaminobenzidine (DAB) solution, stained using hematoxylin, dehydrated, and mounted.

Result evaluation: ten randomly selected high magnification fields in each section were observed under light microscopy by two senior physicians using a double-blind method, and the final results were averaged. PIK3CA results were determined by scoring the staining intensity into unstained (0 score), light yellow (1 score), dark yellow (2 scores), and brown (3 scores) and scoring the percentage of positive cells into ≤1% (0 score), 1%–20% (1 score), 21%–50% (2 scores), and >50% (3 scores). The positive PIK3CA was defined as positive, when the sum of these two scores ≥4, and as negative, when <4. RIP2 is located in the cytoplasm or around the nuclear membrane and is defined as positive when >40%.

### 2.4. Follow-Up

Follow-up was performed by the phone or WeChat APP, and the last follow-up time was January 31, 2022. None of these 68 patients were lost to follow-up. The primary endpoint was overall survival and the second endpoint was disease-free survival. The starting point was set as receiving chemotherapy after diagnosis. The end point of overall survival (OS) was all cause death. The last follow-up time was January 31^st^ 2022.

### 2.5. Statistical Analysis

SPSS 23.0 software was used to perform statistical analysis. Measurement data were expressed as mean ± standard deviation (SD) and test by one-way ANOVA or LSD method. The categorical data were expressed as *n* (%) and tested by Chi-square test. Cox proportional hazard regression was conducted to explore risk factors and hazard ratios. All variables with *P* < 0.20 in univariate analysis were enrolled in multivariate analysis. *P* < 0.05 was considered as statistically significant.

## 3. Results

### 3.1. Clinical Features of DLBCL Patients

**Figure 1:**
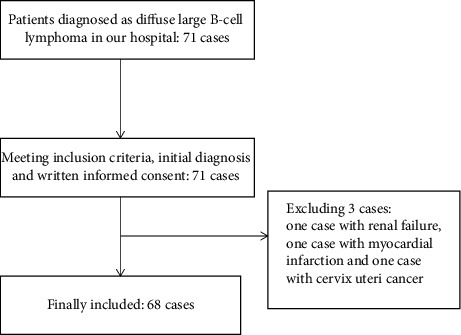
Flowchart of patients' selection.

A total of 68 patients with DLBCL were included in this study (Figure 1), with a mean age of 56.28 ± 10.28 years (23 to 77).

Of these, there were 45 (66.18%) cases with positive VEGF, 44 (64.71%) cases with positive IL-8, 43 (63.42%) cases with positive IL-10, 41 (60.29%) cases with positive PIK3CA, and 42 (61.75%) cases with positive RIP2. There was no difference of VEGF, IL-8, IL-10, PIK3CA, and RIP2 among different subgroups, including age, sex, histology type, and symptoms (*P* > 0.05). There was also no difference of PIK3CA among subgroups, including bone marrow involvement, extranodal involvement, serum LDH level, and number of extranodal sites (*P* > 0.05). The serum level or expression of VEGF, IL-8 and IL-10 were significantly elevated with the increase of Ann Arbor Stage, International Prognostic Index (IPI) scores, Eastern Cooperative Oncology Group (ECOG) scores, serum lactate dehydrogenase (LDH) level, and the number of extranodal sites (all *P* < 0.05). Beside, these serum indexes were significantly higher in patients with the presence of extranodal involvement, but significantly lower in patients with the presence of bone marrow involvement (all *P* < 0.05). The number of patients with positive RIP2 and PIK3CA were significantly higher in patients with more extranodal site, serum LDH level ≥245, high IPI score, ECOG score ≥2, absence of bone marrow involvement, and extranodal involvement (all *P* < 0.05) (Table 1).

### 3.2. Survival Analysis

The mean follow-up time was 39.87 ± 9.23 (24 to 54) months.

The OS of VEGF low expression and high expression groups were 33.12 and 14.28 months, respectively. The OS of PIK3CA low expression and high expression groups were 36.87 and 17.76 months, respectively. The OS of IL-8 low expression and high expression groups were 39.81 and 19.38 months, respectively. The OS of IL-10 low expression and high expression groups were 39.87 and 19.47 months, respectively. The OS of RIP2 low expression and high expression groups were 37.91 and 16.28 months, respectively (all *P* < 0.001).

### 3.3. Univariate Cox Analysis

As shown in Table 2, univariate analysis revealed that Ann Arbor Stage III-IV (HR = 4.302, 95% CI: 1.403–11.098, *P*=0.004), number of extranodal site >1 (HR = 1.573, 95% CI: 1.013–2.761, *P* < 0.001), serum LDH level ≥245 U/L (HR = 1.545, 95% CI: 0.893–2.198, *P*=0.028), ECOG scores ≥2 (HR = 3.297, 95% CI: 1.309–8.076, *P*=0.003), the bone marrow involvement (HR = 1.298, 95% CI: 1.009–1.765, *P*=0.025), extranodal involvement (HR = 1.342, 95% CI: 1.201–1.612, *P* < 0.001), high IL-8 level (HR = 6.048, 95% CI: 4.378–12.981, *P* < 0.001), high IL-10 level (HR = 3.462, 95% CI: 1.254–9.028, *P*=0.013), high VEGF level (HR = 1.026, 95% CI: 0.738–1.329, *P*=0.008), positive PIK3CA (HR = 5.321, 95% CI: 1.096–7.438, *P* < 0.001), and positive RIP2 (HR = 3.189, 95% CI: 1.651–5.034, *P*=0.016) were risk factors for prognosis of DLBCL patients.

### 3.4. Multivariate Cox Analysis

All variables with *P* < 0.20 in univariate analysis were enrolled into multivariate analysis. Multivariate Cox regression revealed that Ann Arbor Stage III-IV (HR = 1.654, 95% CI: 1.234–2.564, *P*=0.009), number of extranodal site >1 (HR = 4.576, 95% CI: 2.289–6.041, *P* < 0.001), serum LDH level ≥245 U/L (HR = 1.674, 95% CI: 1.121–2.675, *P*=0.006), IPI scores 3–5 (HR = 2.276, 95% CI: 2.753–8.976, *P* < 0.001), ECOG scores ≥2 (HR = 2.276, 95% CI: 1.673–4.321, *P*=0.023), bone marrow involvement (HR = 2.429, 95% CI: 1.531–4.652, *P* < 0.001), high expression level of IL-8 (HR = 1.782, 95% CI: 1.021–3.009, *P*=0.034), high expression of IL-10 (HR = 2.179, 95% CI: 1.128–4.098, *P*=0.012), high expression VEGF (HR = 3.041, 95% CI: 1.762–5.091, *P* < 0.001), and positive RIP2 (HR = 1.765, 95% CI: 1.017–3.021, *P*=0.039) were all independent risk factors for the prognosis of DLBCL patients (Table 3).

## 4. Discussion

DLBCL, the most common type of non-Hodgkin lymphoma, lacks specificity in clinical presentation. Although DLBCL receiving first-line R-CHOP-21 chemotherapy obtained a 2-year survival rate of 74.8% and a 10-year survival rate of 36.5%, there were still 30–40% of patients who relapsed [11]. Therefore, it is important to accurately and comprehensively assess the prognosis of patients and to adopt individualized treatment for them. IPI score and gene expression profile-based disease type are accepted method to assess disease prognosis, but not enough to fully explain the reasons that some patients owing poorly response to treatment. Therefore, it is crucial to find novel and effective assessment factors.

PIK3CA gene activation mutations are presented in a variety of malignancies, such as lymphoma, breast, head and neck colorectal, and cervical cancer. Xu et al. indicated that PIK3CA was negatively correlated with the prognosis of DLBCL patients [12]. There were copy number amplification and copy number loss of PI3K (without PIK3R1) and AKT subunits in DLBCL patients, and the copy number variation of PIK3CA was highly correlated with abnormal p110*α* protein expression and subsequent PI3K/AKT pathway activation, which was significant for the prognosis of DLBCL patients [13]. In recent years, with the development of protein monitoring in the diagnosis of disease, serum VEGF test has been a golden standard [14], and caused phosphorylation of Akt and activation of PI3K/Akt/mTOR signaling pathway, suggesting that PIK3CA amplification might be oncogenic. Further *in vitro* experiments confirmed that knockdown of PIK3CA in DLBCL cell lines OCI-LY8 and OCI-LY1 significantly reduced proliferation and promoted apoptosis in a blocking G1 phase manner [15]. Furthermore, Copanlisib, a PI3K inhibitor with *α*/*δ* activity, extremely exhibited high cytotoxicity in all B-cell receptor-dependent DLBCL [16]. On the contrary, there is a close relationship between solid tumor invasion, growth, metastasis, and prognosis with neovascularization, which has become a new method for monitoring tumor progression VEGF, the most important factor regulating neo-angiogenesis, is synthesized by macrophages, vascular endothelial cells, and tumor cells and acts specifically on receptors in vascular endothelial cells in an autocrine or paracrine manner; thus, promoting the growth, migration, proliferation of endothelial cells, the formation of vascular tubular structures, and extracellular matrix degradation [13]. In recent years, the development of medical technology has promoted the application of protein determination in disease diagnosis, and the detection of VEGF levels in serum has become a method for tumor diagnosis [17]. Previous studies showed that invasive metastasis was significantly associated with high expression of VEGF in solid tumors, such as hepatocellular carcinoma and colorectal cancer [17, 18]. Guo et al. [19] and Deng et al. [20] confirmed that VEGF levels were closely associated with DLBCL, serum VEGF levels were highly expressed in patients with DLBCL, and correlated with LDH levels and histological types. It has been used as an important indicator for evaluating the prognosis of DLBCL due to the lack of clear diagnostic and prognostic indicators for DLBCL [21]. IL-8 is one of the proangiogenic chemokines and plays an important role in the interaction between tumor-associated macrophages and tumor cells [22]. DLBCL cells could recruit neutrophils in the blood by secreting IL-8 and express the proliferation-inducing ligand, which in turn leads to immune escape of tumor cells [23]. IL-10, a pleiotropic cytokine that has a bidirectional regulatory immunomodulatory effect in the organism, can promote immune response by acting on CD8^+^ T cells and negatively regulates the immune response by acting on regulatory T cells [24]. It was noted that high expression of IL-10 mRNA in tumor tissues of patients with aggressive B-cell lymphoma could be an important indicator for prognosis of DLBCL [25]. RIP2 is a serine/threonine kinase with a caspase activation and recruitment structural domain at its carboxyl terminus, known for its role in inflammation and immunity, and is important for maintaining protein stability [26]. RIP2 is closely associated with the occurrence, development, and prognosis of tumors such as bladder cancer, triple-negative breast cancer, and oral phosphorylated cell carcinoma. The survival rate of DLBCL patients in the RIP2-positive group was lower than that of the PIP2-negative group, suggesting that RIP2 expression might be associated with poor prognosis in DLBCL [9]. Previous study has proved that RIP2 is associated with the activation of NF-*κ*B, c-Jun N-terminal kinase, extracellular signal-regulated kinase, and mitogen-activated protein kinase p-38 pathways [27]. In DLBCL, aberrant activation of the NF-*κ*B is thought to be closely related to tumor cells, patient survival, and drug resistance, and inhibiting the NF-*κ*B activity significantly enhances chemotherapeutic drug-induced apoptosis [9].

In this study, the results showed significant differences of VEGF, IL-8, IL-10, and RIP2 between subgroups for Ann Arbor Stage, IPI scores, ECOG scores, the bone marrow involvement, extranodal involvement, serum LDH level, and the number of extranodal site (all *P* < 0.05), suggesting the possible connections of VEGF, IL-8, and IL-10 levels to the above clinical features. Further Cox proportional hazard regression analysis of prognosis showed that that Ann Arbor stage (stage III-IV), the number of extranodal site (>1), serum LDH level (≥245 U/L), IPI scores (3–5), ECOG scores (≥2), and the bone marrow involvement were risk factors for the prognosis of DLBCL patients. More importantly, high expression of VEGF, IL-8, IL-10, and positive PIK3CA and RIP2, were also risk factors for the prognosis of DLBCL patients, and the possible mechanisms are as follows: firstly, angiogenesis is extremely important for the growth, malignancy and metastasis of DLBCL. VEGF, which can act on vascular endothelial mitosis and induce the neovascularization formation, is the most potent cytokine contributing to tumor angiogenesis [19] and is a vascular proliferation factor with high specificity in the formation of DLBCL vessels. In addition, the vascular induced by VEGF has high permeability, and the plasma proteins during circulation easily penetrate into the extracellular matrix, providing a good growth environment for endothelial cells and fibroblasts to form a vascular-rich tumor stroma, thus providing conditions for tumor metastasis and infiltration. Secondly, IL-8 induces neutrophils to accumulate and infiltrate at lymphoma sites, aggravating the inflammatory response of lymphoma [25]. Thirdly, the weak immunogenicity of IL-10, and the confinement and decorative effect of tumor antigens, together causes immune escape phenomenon due to the inability of the organism to recognize tumor antigens. In addition, IL-10 can act as a potent immunosuppressant to regulate the function of B lymphocytes, which in turn affects the developmental process of DLBCL [9]. Finally, RIP2 is able to activate the nonclassical NF-*κ*B pathway in human Burkitt lymphoma cells, which may be one of the mechanisms by which RIP2 affects the prognosis of DLBCL patients [28].

There were also some limitations in this study. First, the sample size of this study was small and not representative enough, and the sample size should be expanded and the follow-up period should be extended in the future study. Second, the main tissues tested in this study for IHC were tumor tissues, and paraneoplastic tissues were not included to compare the differences of VEGF, IL-8, IL-10, PIK3CA, and RIP2. Third, this study was a retrospective analysis, and a prospective study should be used in the future to further confirm the results.

## 5. Conclusion

In conclusions, advanced Ann Arbor Stage, higher number of extranodal site, high serum LDH level, high IPI score, high ECOG score, bone marrow involvement, high VEGF, high IL-8, high IL-10, and positive RIP2 were all risk factors of DLBCL overall survival time.

## Figures and Tables

**Table 1:** Comparison of VEGF, IL-8, IL-10, PIK3CA, and RIP2 in DLBCL patients with different clinical features.

Clinical features	*n*	VEGF (pg/mL)	IL-8 (pg/ml)	IL-10 (pg/ml)	PIK3CA positive (*n*)	RIP2 positive (*n*)
Age (years)						
<60	30	554.78 ± 73.19	26.98 ± 4.21	42.78 ± 10.29	21	24
≥60	38	578.92 ± 81.12	26.01 ± 4.19	45.93 ± 10.34	20	18
*χ*^2^/*t*	—	−1.272	0.946	−1.250	0.716	1.249
*P*	—	0.208	0.348	0.216	0.398	0.364

Gender						
Male	37	553.23 ± 69.98	24.93 ± 7.34	44.01 ± 10.28	19	17
Female	31	563.28 ± 74.91	26.87 ± 8.27	46.93 ± 11.23	22	25
*χ*^2^/*t*	—	−0.571	−1.025	−1.118	0.178	2.412
*P*	—	0.570	0.309	0.268	0.673	0.120

Ann Arbor stage						
I	15	531.81 ± 36.98	29.32 ± 5.03	7.93 ± 2.01	8	8
II	17	589.77 ± 32.82	22.13 ± 5.18	27.63 ± 2.31	12	13
III	22	609.73 ± 33.98	15.78 ± 3.32	53.29 ± 6.93	8	7
IV	14	623.87 ± 34.93	9.83 ± 2.32	71.29 ± 8.31	13	14
*F*	—	20.981	62.297	370.785	4.192	10.954
*P*	—	<0.001	<0.001	<0.001	0.241	0.012

Symptoms						
Absence	37	547.91 ± 47.39	25.97 ± 5.32	45.92 ± 10.29	19	18
Presence	31	583.89 ± 52.93	27.22 ± 5.21	48.21 ± 10.32	22	24
*χ*^2^/*t*	—	−1.399	0.974	−0.913	0.494	2.412
*P*	—	0.166	0.334	0.365	0.482	0.120

Extranodal site						
≤1	37	556.28 ± 68.28	20.97 ± 4.31	42.83 ± 10.23	9	10
>1	31	597.92 ± 69.13	29.92 ± 4.23	49.13 ± 10.34	32	32
*χ*^2^/*t*	—	2.490	−8.601	−2.517	25.805	23.048
*P*	—	0.015	<0.001	0.014	<0.001	<0.001

Serum LDH level (U/L)						
<245	32	557.92 ± 53.21	21.23 ± 1.43	43.21 ± 10.37	10	11
≥245	36	598.78 ± 55.18	27.89 ± 1.34	49.39 ± 10.76	31	31
*χ*^2^/*t*	—	−2.992	−19.821	−2.321	21.048	18.319
*P*	—	0.004	<0.001	0.024	<0.001	<0.001

IPI scores						
0-1	13	521.98 ± 32.19	29.48 ± 3.07	8.38 ± 10.23	2	1
2	19	538.91 ± 36.27	22.96 ± 4.45	29.87 ± 11.34	8	7
3	22	568.92 ± 35.28	16.89 ± 3.37	52.29 ± 13.29	19	20
4-5	14	587.38 ± 37.92	12.38 ± 3.72	69.89 ± 14.43	12	14
*F*	—	10.022	56.705	65.52	23.602	37.672
*P*	—	<0.001	<0.001	<0.001	<0.001	<0.001

ECOG scores						
0-1	46	547.98 ± 34.98	28.93 ± 6.28	10.87 ± 1.93	19	20
≥2	22	601.98 ± 35.78	19.49 ± 8.94	67.92 ± 13.29	22	22
*χ*^2^/*t*	—	−5.912	5.035	−28.716	12.168	20.133
*P*	—	<0.001	<0.001	<0.001	<0.001	<0.001

Bone marrow involvement						
Presence	11	535.98 ± 67.93	12.88 ± 3.98	40.93 ± 10.24	11	10
Absence	57	586.91 ± 63.29	33.83 ± 4.34	48.93 ± 10.32	30	32
*χ*^2^/*t*	—	−2.416	−14.838	−2.357	8.642	4.786
*P*	—	0.018	<0.001	0.021	0.003	0.041

Extranodal involvement						
Presence	19	588.89 ± 76.98	17.91 ± 3.92	49.98 ± 10.37	16	18
Absence	49	543.78 ± 73.28	30.38 ± 3.89	43.39 ± 10.24	26	24
*χ*^2^/*t*	—	2.246	−11.836	2.373	5.625	12.138
*P*	—	0.028	<0.001	0.021	0.018	<0.001

Hans						
GCB	31	582.91 ± 71.29	25.99 ± 4.88	45.19 ± 12.19	17	19
Non-GCB	37	564.39 ± 75.38	27.98 ± 4.99	49.38 ± 12.39	24	23
*χ*^2^/*t*	—	1.034	−1.654	−1.399	0.709	0.005
*P*	—	0.305	0.102	0.166	0.461	0.941

DLBCL, diffuse large B-cell lymphoma; LDH, lactate dehydrogenase; GCB, germinal center B-cell; IPI, international prognostic index; ECOG, eastern cooperative oncology group; PIK3CA, phosphatidylinositol-4,5-bisphosphate 3-kinase catalytic subunit alpha; VEGF, vascular endothelial growth factor; IL, interleukin; RIP2, receptor-interacting protein-2.

**Table 2:** Regression analysis of the univariate Cox proportional hazard model for overall survival.

Clinical features	*b*	*S*_*b*_	Wald *χ*^2^	HR	95% CI	*P* value
Age (years)						
<60 vs. ≥60	0.619	0.567	1.201	1.867	0.603–5.672	0.269

Gender						
Male vs. female	−0.287	0.503	0.846	0.631	0.218–1.654	0.347

Ann Arbor Stage						
I, II vs. III, IV	1.376	0.529	7.389	4.302	1.403–11.098	0.004

B symptoms						
Presence vs. absence	0.417	0.387	1.081	1.509	0.683–3.201	0.287

Extranodal site						
≤1 vs. >1	0.821	0.267	4.762	1.573	1.013–2.761	<0.001

Serum LDH level (U/L)						
<245 vs. ≥245	0.298	0.331	2.786	1.545	0.893–2.198	0.028

IPI scores						
0–2 vs. 3–5	−0.401	0.603	0.488	0.675	0.209–2.061	0.453

ECOG scores						
0-1 vs. ≥2	1.109	0.434	7.135	3.297	1.309–8.076	0.003

Bone marrow involvement						
Presence vs. absence	0.351	0.292	3.091	1.298	1.009–1.765	0.025

Extranodal involvement						
Presence vs. absence	0.318	0.102	8.659	1.342	1.201–1.612	<0.001

Hans						
GCB vs. non-GCB	0.371	0.529	0.469	1.429	0.499–4.091	0.489

VEGF level						
Low vs. high	0.221	0.318	0.352	1.026	0.738–1.329	0.008

PIK3CA						
Negative vs. positive	1.731	0.857	2.372	5.321	1.096–7.438	<0.001

IL-8 level						
Low vs. high	1.903	0.937	9.682	6.048	4.378–12.981	<0.001

IL-10 level						
Low vs. high	1.298	0.518	5.703	3.462	1.254–9.028	0.013

RIP2						
Negative vs. positive	1.176	0.706	7.902	3.189	1.651–5.034	0.016

DLBCL, diffuse large B-cell lymphoma; LDH, lactate dehydrogenase; GCB, germinal center B-cell; IPI, international prognostic index; ECOG, eastern cooperative oncology group; PIK3CA, phosphatidylinositol-4,5-bisphosphate 3-kinase catalytic subunit alpha; VEGF, vascular endothelial growth factor; IL, interleukin; RIP2, receptor-interacting protein-2; HR, hazard ratio; CI, confidence interval.

**Table 3:** Multivariate Cox proportional hazard regression analysis of prognosis.

Clinical features	*b*	*S*_*b*_	Wald *χ*^2^	HR	95% CI	*P* value
Ann Arbor Stage						
I, II vs. III, IV	0.575	0.206	6.231	1.654	1.234–2.564	0.009

B symptoms						
Presence vs. absence	0.765	0.409	3.721	2.134	1.064–3.342	0.062

Extranodal site						
≤1 vs. >1	1.532	0.387	23.954	4.576	2.289–6.041	<0.001

Serum LDH level (U/L)						
<245 vs. ≥245	0.621	0.231	7.674	1.674	1.121–2.675	0.006

IPI scores						
0–2 vs. 3–5	1.813	0.874	13.276	5.231	2.753–8.976	<0.001

ECOG scores						
0-1 vs. ≥2	1.033	0.268	4.409	2.276	1.673–4.321	0.023

Bone marrow involvement						
Presence vs. absence	1.039	0.159	14.762	2.429	1.531–4.652	<0.001

Extranodal involvement						
Presence vs. absence	1.896	0.127	4.201	6.721	2.187–15.543	0.431

VEGF level						
Low vs. high	1.109	0.0281	14.289	3.041	1.762–5.091	<0.001

PIK3CA						
Negative vs. positive	1.659	0.673	2.369	5.187	1.398–8.971	0.019

IL-8 level						
Low vs. high	0.573	0.268	4.291	1.782	1.021–3.009	0.034

IL-10 level						
Low vs. high	0.765	0.352	5.728	2.179	1.128–4.098	0.012

RIP2						
Negative vs. positive	0.567	0.278	4.163	1.765	1.017–3.021	0.039

DLBCL, diffuse large B-cell lymphoma; LDH, lactate dehydrogenase; GCB, germinal center B-cell; IPI, international prognostic index; ECOG, eastern cooperative oncology group; PIK3CA, phosphatidylinositol-4,5-bisphosphate 3-kinase catalytic subunit alpha; VEGF, vascular endothelial growth factor; IL, interleukin; RIP2, receptor-interacting protein-2; HR, hazard ratio; CI, confidence interval.

## Data Availability

The datasets generated and analyzed during the current study are available from the corresponding author on reasonable request.

## Abbreviations

DLBCL: Diffuse large B-cell lymphoma
DAB: Diaminobenzidine
ECOG: Eastern Cooperative Oncology Group
IL: Interleukin
IPI: International prognostic index
GCB: Germinal center B-cell
LDH: Lactate dehydrogenase
PBS: Phosphate buffer saline
PIK3CA: Phosphatidylinositol-4,5-bisphosphate 3-kinase catalytic subunit alpha
RIP2: Receptor-interacting protein-2
VEGF: Vascular endothelial growth factor.

## References

[1] Tun A. M., Ansell S. M. (2020). Immunotherapy in Hodgkin and non-Hodgkin lymphoma: i. *Cancer Treatment Reviews*.

[2] Narkhede M., Cheson B. D. (2020). Copanlisib in the treatment of non-Hodgkin lymphoma. *Future Oncology*.

[3] Ow K. V., Brant J. M. (2021). Non-hodgkin lymphoma: examining mycosis fungoides and sézary syndrome in the context of Oncology nursing. *Clinical Journal of Oncology Nursing*.

[4] Bojarczuk K., Wienand K., Chapuy B. (2020). Molecular classification of large B-cell non-hodgkin lymphoma. *Cancer Journal*.

[5] Xu W., Berning P., Lenz G. (2021 Sep 30). Targeting B-cell receptor and PI3K signaling in diffuse large B-cell lymphoma. *Blood*.

[6] Sang W., Zhou H., Qin Y. (2021). Risk stratification model based on VEGF and International Prognostic Index accurately identifies low-risk diffuse large B-cell lymphoma patients in the rituximab era. *International Journal of Hematology*.

[7] Wright G. W., Huang D. W., Phelan J. D. (2020). A probabilistic classification tool for genetic subtypes of diffuse large B cell lymphoma with therapeutic implications. *Cancer Cell*.

[8] Stirm K., Leary P., Bertram K. (2021). Tumor cell-derived IL-10 promotes cell-autonomous growth and immune escape in diffuse large B-cell lymphoma. *OncoImmunology*.

[9] Wang X. C., Ding K. Y., Wang X. Q., Wang Z. H., Wang J (2018). Expression and clinical prognostical significance of RIP2 in diffuse large B cell lymphoma. *Zhongguo Shi Yan Xue Ye Xue Za Zhi*.

[10] Guo F. F. (2018). *Analysis of Prognostic Factors in Patients with Diffuse Large B-Cell Lymphoma*.

[11] Manji F., Puckrin R., Stewart D. A. (2021). Novel synthetic drugs for the treatment of non-Hodgkin lymphoma. *Expert Opinion on Pharmacotherapy*.

[12] Xu Z. Z., Xia Z. G., Wang A. H. (2013). Activation of the PI3K/AKT/mTOR pathway in diffuse large B cell lymphoma: clinical significance and inhibitory effect of rituximab. *Annals of Hematology*.

[13] Cui W., Cai Y., Wang W. (2014). Frequent copy number variations of PI3K/AKT pathway and aberrant protein expressions of PI3K subunits are associated with inferior survival in diffuse large B cell lymphoma. *Journal of Translational Medicine*.

[14] Ma M. F., Yu L., Ma L. P. (2018). PIK3CA amplification and PTEN deletion in invasive B-cell lymphoma and their clinicopathological significance[J]. *Chinese Journal of Clinical Oncology*.

[15] Cui W., Zheng S., Liu Z. (2017). PIK3CA expression in diffuse large B cell lymphoma tissue and the effect of its knockdown in vitro. *OncoTargets and Therapy*.

[16] Bojarczuk K., Wienand K., Ryan J. A. (2019). Targeted inhibition of PI3K*α*/*δ* is synergistic with BCL-2 blockade in genetically defined subtypes of DLBCL. *Blood*.

[17] Ma X. F., Lin Z. W. (2020). Expression and clinical significance of serum MIF and VEGF in patients with primary liver cancer [J]. *Journal of Hepatobiliary Surgery*.

[18] Shen S., Xi Y. F. (2020). The diagnostic value of MSCT combined with VEGF in colorectal cancer [J]. *Chinese Journal of Gastroenterology and Hepatology*.

[19] Guo Q., Wang J. J., Li F. (2013). Expressions of VEGF and CXCR4 in diffuse large B cell lymphoma and their clinical significances. *Zhongguo Shi Yan Xue Ye Xue Za Zhi*.

[20] Deng C., Wu S., Zhang L. (2018). Role of monocyte tissue factor on patients with non-small cell lung cancer. *Clinical Research J*.

[21] Jiang Y. J., Zhu G. H., He Y. (2019). Clinical significance of TF and VEGF expressions on peripheral CD14 positive monocytes in patients with diffuse large B cell lymphoma. *Zhongguo Shi Yan Xue Ye Xue Za Zhi*.

[22] Tang M. (2011). *Clinical Significance of the Quantity of Tumor Associated Macrophages and the Expression of IL-6、IL-8 in Diffuse Large B-Cell Lymphoma and Follicular Lymphoma*.

[23] Pontarini E., Murray-Brown W. J., Croia C. (2020). Unique expansion of IL-21+ Tfh and Tph cells under control of ICOS identifies Sjögren’s syndrome with ectopic germinal centres and MALT lymphoma. *Annals of the Rheumatic Diseases*.

[24] Ferreri A. J. M., Calimeri T., Lopedote P. (2021). *MYD88* L265P mutation and interleukin-10 detection in cerebrospinal fluid are highly specific discriminating markers in patients with primary central nervous system lymphoma: results from a prospective study. *British Journal of Haematology*.

[25] Xie Y., Li C. P., Tan L. (2018). Changes and significance of IL-11 and IL-10 mRNA expression in diffuse large B-cell lymphoma. *Shandong Medical Journal*.

[26] Rahman M. A., Sundaram K., Mitra S., Gavrilin M. A., Wewers M. D (2014). Receptor interacting protein-2 plays a critical role in human lung epithelial cells survival in response to fas-induced cell-death. *PLoS One*.

[27] Sun J., Wang L. C. S., Fridlender Z. G. (2011). Activation of mitogen-activated protein kinases by 5, 6-dimethylxanthenone-4-acetic acid (DMXAA) plays an important role in macrophage stimulation. *Biochemical Pharmacology*.

[28] Cai X., Wang M., Kong H. (2013). Prokaryotic expression, purification and functional characterization of recombinant human RIP2. *Molecular Biology Reports*.

